# Gluten and Wheat in Women’s Health: Beyond the Gut

**DOI:** 10.3390/nu16020322

**Published:** 2024-01-22

**Authors:** Francesca Manza, Lisa Lungaro, Anna Costanzini, Fabio Caputo, Umberto Volta, Roberto De Giorgio, Giacomo Caio

**Affiliations:** 1 Department of Translational Medicine, University of Ferrara, 44121 Ferrara, Italy; mnzfnc@unife.it (F.M.); anna.costanzini@unife.it (A.C.); fabio.caputo@unife.it (F.C.); dgrrrt@unife.it (R.D.G.); 2 Department of Medical and Surgical Sciences, University of Bologna, 40138 Bologna, Italy; umberto.volta@unibo.it; 3 Mucosal Immunology and Biology Research Center, Massachusetts General Hospital—Harvard Medical School, Boston, MA 02114, USA

**Keywords:** gluten-free diet, wheat, women, autoimmunity, fibromyalgia, endometriosis, non-celiac gluten sensitivity, autoimmune thyroiditis, irritable bowel syndrome, exclusion diet

## Abstract

Since the rise of awareness of gluten/wheat-related disorders in the academic and clinical field in the last few decades, misinformation regarding the gluten-free diet (GFD) and its impact on health has been spreading among the general population. Despite the established link between gluten and celiac disease (CD), where a GFD is mandatory to reach clinical and histological remission, things are more complicated when it comes to non-celiac gluten/wheat sensitivity (NCGWS) and other autoimmune/dysimmune disorders. In the last conditions, a beneficial effect of gluten withdrawal has not been properly assessed, but still is often suggested without strong supporting evidence. In this context, women have always been exposed, more than men, to higher social pressure related to nutritional behaviors and greater engagement in controlling body weight. With this narrative review, we aim to summarize current evidence on the adherence to a GFD, with particular attention to the impact on women’s health.

## 1. Wheat and Gluten

Wheat is a carbohydrate source appreciated for its versatility and widely diffused all over the globe. Moreover, wheat is a cheap source of proteins, with a long shelf life; it is ready to use, and its combination with legumes has a complete amino acidic profile [1]. Wheat is a complex mixture of proteins, starches, fibers, and micronutrients, which confers its rheological and culinary properties, but also the prerequisites for wheat allergy (WA), celiac disease (CD), and non-celiac gluten/wheat sensitivity (NCGWS).

Gluten is a storage protein of wheat, rye, and barley. It is a complex mixture of prolamins, particularly rich in proline and glutamine. These amino acids cause the twisting of the protein chain, resulting in numerous kinks and folds. The mixture of gluten and water forms a cohesive compound: during the kneading process, the chains bond together through disulfide, ionic, and H-links. This gives gluten its fascinating ability to form elastic, balloon-like films that hold gas produced during the rising and baking process [2].

Gluten is insoluble in water and in most neutral solvents, despite its ability to absorb water. Gluten components can be separated in alcohol into soluble gliadins (THREE major forms: α-, γ-, and ω-gliadin) and insoluble glutenins.

The proline- and glutamine-rich residues, gliadins, which are resistant to human protease digestion, are the trigger for CD in genetically predisposed subjects, along with an increased intestinal permeability [3]. 

Regarding WA, the advance made in molecular diagnostic tools has allowed for the identification of wheat allergogenic proteins involved. In particular, a lipid transfer protein (LTP, Tri a 14), omega-5-gliadin (Tri a 19) and amylase trypsin inhibitor family (ATIs) are the only molecular targets of the IgE-mediated response available as a diagnostic tool [4]. 

The pathophysiological mechanisms become less clear when it comes to NCGWS. It is still debated whether the component linked to exacerbation of intestinal and extraintestinal symptoms of NCGWS is gluten [5] or other components of wheat such as ATIs or FODMAPs (fermentable oligo-, di-, and monosaccharide and polyol) [6,7]. FODMAPs can cause bloating in different functional gastrointestinal disorders.

However, in CD, a lifelong gluten-free diet (GFD) is the only effective therapy, ameliorating symptoms and restoring intestinal morphology, in the vast majority of patients. Similarly, a GFD is beneficial in NCGWS, but it is still debated if the latter could be identified as a reversible condition [8], and therefore if the adherence to a GFD is temporary.

## 2. Wheat as a Trigger of Immune Response: The Leaky Gut

It has become increasingly clear that the human intestine is the largest surface where the interplay between ourselves and the external environment takes place. More specifically, Fasano analyzed the mechanisms behind intestinal permeability and molecular trafficking between the intestinal lumen and the submucosa [9]. Under normal circumstances, antigenic and molecular trafficking is strictly regulated by several processes that ensure homeostasis of the process and immune tolerance. In this context, zonulin has a pivotal role in the regulation of gut permeability, acting as a modulator of barrier integrity. Its production is mainly triggered by bacteria overgrowth [10] and gluten [11]. Zonulin secretion leads to an increase in gut permeability, removing the zonula occludens 1, a component of intercellular tight junction. The increase in antigenic trafficking between the lumen and the lamina propria, often referred as “the leaky gut”, has been implicated in the pathogenesis of many chronic inflammatory diseases.

Clemente et al. studied the effects of gliadin on zonulin release in intestinal epithelial cells in vitro [5]. Enterocytes secrete increasing levels of zonulin after gliadin exposure, but zonulin levels return to baseline after 30 min. Despite this effect also being seen in healthy control tissue, it is upregulated in CD patients, associated with an increased baseline gut permeability and an increased amplitude and duration of gluten-induced zonulin release [11].

Thus, the overproduction of zonulin seems to have a role in the mechanisms regulating the immune system, through the tuning of intestinal permeability [5]. Since zonulin production is related to gliadin exposure, the exclusion of gluten-containing products has often been claimed to be beneficial.

## 3. What Is a Gluten-Free Diet?

A GFD consists of the avoidance of gluten-containing foods (e.g., wheat, barley, spelt, kamut, rye, triticale). The diet is usually implemented with gluten-free (GF) sources of carbohydrates (e.g., rice, quinoa, corn, buckwheat, legumes) and/or the consumption of industrial GF products. 

Intriguingly, the European market of GF products is expected to grow from USD 3.28 billion in 2023 to USD 5.37 billion by 2028 [12]. Given that the variety of GF products as well as the ease to access them is consequently increasing, the concerns related to the healthiness of a GFD cannot be neglected. Gluten-free products are often richer in saturated fat, sugar, and salt [13,14], and contain lower levels of proteins, fiber, and vitamins [15]; a GFD has been associated with an increased risk of deficiency of micronutrients, hyperlipidemia, hyperglycemia, and coronary artery disease [16].

Despite these unequivocal findings, among the reasons leading healthy subjects to pursue a GFD, we can find weight control, the perceptions that this dietary regimen is healthier, and symptoms triggered by gluten ingestion. The majority of consumers are women, often with other food intolerances (e.g., lactose) [17,18]. A few studies suggest that individuals avoiding gluten have an overall better nutritional profile, but this is probably due to a specific attention in maintaining a healthier lifestyle, avoiding processed foods, and implementing fruit and vegetable consumption [19,20].

Another aspect worth of consideration is how a GFD impacts on gut microbiota. Current evidences suggest that a GF regime can reduce bacterial richness while affecting gut microbiota composition, especially in healthy subjects, causing a reduction in probiotic species such as Bifidobacteria, in favor of opportunistic pathogens, e.g., Enterobacteriaceae and *Escherichia coli* [3,21].

Therefore, with this narrative review, we would like to summarize current evidence regarding the application of a GFD, focusing on the women’s point of view and diseases with an higher incidence in females.

## 4. Methods

We conducted PubMed, EMBASE, MEDLINE, and ScienceDirect search, including original papers from January 2000 to October 2023. Literature was screened using the following keywords: “gluten free diet”, “gluten free diet AND women”, “gluten AND autoimmunity”, “endometriosis AND gluten”, “gluten free diet AND women”, “gluten free diet AND female”, “women AND celiac disease”, “women AND non celiac gluten sensitivity”, “gluten AND thyroiditis”, “gluten AND fibromyalgia”, “gluten AND endometriosis”; related results were also considered. Only papers (1) consistent with the topic, (2) written in English or (3) Italian, and (4) available in full text were included in this narrative analysis. Records were screened independently by L.L and F.M. and subsequently reviewed by the other authors. Duplicates, papers with no original data, and incomplete or unclear outcomes were excluded.

## 5. Celiac Disease

CD is an autoimmune disorder, affecting around 1–1.5% of the population, an increasing percentage, especially in Western countries [22,23]. A diagnosis of CD is 1.5–2 times more common in female than males [24,25]. CD is often associated with different autoimmune diseases, including dermatitis herpetiformis, type 1 diabetes mellitus, Hashimoto’s thyroiditis, selective IgA deficiency, alopecia areata, Addison’s disease, connective tissue diseases (mainly Sjogren’s syndrome), and hepatic autoimmune diseases, but also neurological diseases (cerebellar ataxia, peripheral neuropathy, epilepsy) [26,27,28]. Diagnosing CD in the presence of those conditions is pivotal since following a GFD could lead to gastrointestinal symptom resolution, prevent complications, and improve some of the CD-associated diseases [22,29]. Jacobsson et al. carried out a qualitative research regarding the condition of women with CD in Sweden. The study highlighted how women’s perception of food safety, food risk management, social inclusion, and the ability to be in control of food-related situations should not be underestimated. In a context where the goal of patients with CD is to experience normality in life, coping mechanisms are necessary. Healthcare professionals should support patients in everyday challenges [30]. A study by Hallert et al. [31] digs into gender-related differences in people diagnosed with CD, and compares these aspects with the male counterpart. Women living with CD report poorer health-related quality of life (QoL) than men do, related specifically to (1) the permanence of bowel symptoms despite following a GFD, (2) a depressive–anxious behavior related to feelings of being different (identification), and (3) a passive–adaptive psychological attitude towards the disease (acceptance) [32].

Moreover, CD is known to be associated with conditions influencing female reproductive system and pregnancy outcomes. In particular, untreated CD is related to amenorrhea, puberty delay, spontaneous abortion, and stillbirth, despite no gynecological differences between already-diagnosed CD and healthy controls being found [33,34]. A higher prevalence of CD in unexplained infertility was demonstrated in different studies [35,36]. Several studies suggest that following a rigorous GFD might be beneficial in the improvement of fertility rates, despite larger and higher-quality studies being needed to reach higher significancy [37,38].

Celiac women are at a higher risk of osteoporosis as a consequence of nutrient malabsorption and indirect hormonal effects (such as early menopause, amenorrhea), but a GFD alone is effective in the improvement of bone mass density (BMD) [39,40,41].

Overall, a strict adherence to a GFD is the only available and effective treatment for CD and its complications [22,42,43]. Dietetic support is mandatory to avoid malnutrition and to decide whether supplementation is needed. The phenotype of CD patients is changing with the increasing prevalence of obesity. Patients can be overnourished while still having micronutrient deficiencies [44].

However, it must be underlined that 10–19% of CD patients might present persistent symptoms despite following a GFD, mainly because of unintentional cross-contamination. Other subgroups might be classified as late responders, IBS, or refractory [45,46,47].

## 6. Non-Celiac Gluten/Wheat Sensitivity (NCGWS) and Irritable Bowel Syndrome (IBS)

NCGWS is a syndrome characterized by intestinal and extraintestinal symptoms related to the ingestion of gluten-/wheat-containing food, where CD and WA have already been excluded. For the sake of clarity, from now on, “wheat” will be the word used to include all the components that might trigger a symptomatology. A double-blind placebo-controlled challenge (DBPC) using wheat triggering intestinal and extraintestinal symptoms is nowadays considered the gold standard for diagnosis [48]. NCGWS prevalence has not been adequately established, especially due to the lack of specific markers, the difficulties related to the application of a DBPC in real practice, and the uncertain etiology of reported symptoms [49]. 

The GFD is gaining popularity as a healthy diet thanks to media hype [50]. Gluten intolerance is more frequently reported by women, who are also more likely to avoid wheat-containing foods [17]. The motivations behind these self-made choices are mainly (1) the belief that a GFD is healthier and (2) that a GFD helps in controlling body weight. 

However, exclusionary diets (i.e., diets that completely exclude certain categories of foods, such as food containing gluten) may cause health problems. Very often, a nutritional imbalance or deficiency favors the onset of physical pathologies, such as osteopenia. A study by Carroccio et al. [51] investigated the prevalence of osteopenia, comparing CD (8 males, 42 females), IBS (10 males, 55 females) and NCGWS (12 males, 63 females) patients. NCGWS patients had significantly lower BMD than IBS controls, both at the lumbar spine and at the femoral neck (*p* < 0.0001), whereas CD patients had the lowest. These data are meaningful, since the study sample included mainly females, mostly premenopausal. Moreover, the correlation of the results with BMI and hemoglobin confirmed the hypothesis of malnutrition. Indeed, the dietary calcium intake in NCGWS patients was much lower than the recommended dose of 1000 mg/day, probably due to the presence of multiple food sensitivity (including diary), which implies additional dietary restriction. In this regard, a DBPC with cow’s milk proteins was performed in all NCGWS cases, and 30 out of 75 were positive. Globally, these findings highlight the uncertainty of the pathogenesis of osteopenia in NCGWS patients. Etiological factors can be found in malnutrition, in an unbalanced diet or in an equal contribution of the two. However, the results strengthen the need of a nutritional consultation at the time of diagnosis of NCGWS.

Interestingly, in a study by Mansueto et al. [52], the incidence of autoimmune disorders (AD) is higher in NCGWS (25%) than IBS patients (*p* = 0.05) and healthy controls (*p* = 0.002). AD was positively associated with female sex, older age at NCGWS diagnosis, duodenal lymphocytosis, and eosinophilic infiltration. The authors hypothesized that the longer the gluten exposure, the stronger the autoimmune reactivity, triggered by a local inflammation (sustained by the mild inflammation—Marsh 1—found in the biopsies), that may cause an increased permeability and lastly the development of AD. In this study, 71.4% of NCGWS patients had positivity of serum antinuclear antibodies (ANA).

NCGWS and IBS are two clinical entities that are often difficult to distinguish. IBS, which has been defined as a “disorder of gut-brain interaction” [53], is diagnosed relying on the “Rome IV criteria” [54], and it is often responsive to many dietary exclusions, such as FODMAPs. Indeed, the avoidance of FODMAP-rich foods significantly reduces bloating and abdominal pain compared to a standard diet [55,56]. Gastrointestinal symptoms, related to the overdistention of the intestinal lumen, are often common in both NCGWS and IBS patients. Wheat contains fructans in variable percentages (ranging from 1.5% in with flour to 3.7% in barn) [57,58], and it is therefore excluded in the low-FODMAP diet. However, the fructan content is variable, as it depends on the preparation and the specific recipe of the products. For this reason, affirming that GF products are always lower in their fructan content may be inaccurate [59]. Usually, NCGWS patients can clearly point out gluten as being responsible for their symptomatology, whereas IBS patients cannot identify a single dietary compound that is causally related with their disorders [59]. Yet, in a comparative study carried out in the UK, 20% of patients with self-reported NCGWS fulfill IBS Rome III criteria, concluding that NCGWS and IBS are often associated conditions [60]. The presence of extraintestinal symptoms in NCGWS is a distinctive feature. Manifestations such as fatigue, skin rashes, and joint and muscle pain are shortly relieved after gluten avoidance. Furthermore, half of NCGWS patients test positive for IgG antigliadin antibodies, which disappear quickly after GFD [61].

## 7. Fibromyalgia

Fibromyalgia (FM) is a rheumatologic condition characterized by chronic pain, fatigue, sleep mood disturbances, and cognitive impairment. FM affects up to 3% of the American population, with a female-to-male ratio of 2:1. Its management and diagnosis is often challenging for both patients and physicians, resulting in a poor QoL [62]. Despite there being no reference regarding diet therapy in the most recent EULAR guidelines (2016), a review by Aman et al. [63] analyzes different nonpharmacological therapeutic options used by FM patients, including GFD. The rationale behind the adherence to a gluten-free regimen is that there is often an overlap of gastrointestinal symptoms in FM and gluten-related disorders. 

Different studies evaluated the relationship between GFD and the improvement of FM symptoms and FM with regard to QoL (Table 1). Rodrigo et al. [64] registered a slight but significant improvement in FM and IBS symptoms in a subgroup of patients with duodenal intraepithelial lymphocytosis. On the other side, Slim et al. [65] found no difference when comparing a GFD approach to a hypocaloric diet in terms of improvement of gastrointestinal symptomatology; nevertheless, these approaches are both well-tolerated. GFD might be beneficial only in a subset of FM patients, specifically with intraepithelial lymphocytosis [66]. We underline that extraintestinal symptoms of NCGWS include FM-like symptoms. 

Overall, this evidence suggests a cautious approach when proposing GFD indiscriminately to FM patients [67]; nonetheless, gluten avoidance might be beneficial in a subset of patients, whether they are NCGWS with FM-like symptoms or FM with an increased intraepithelial lymphocyte count. More studies specifically involving a gluten challenge might be decisive for a better understanding of this phenomenon. 

**Table 1:** Experimental design studies relating fibromyalgia and gluten-free diet.

Authors	Type of Study	Duration of GFD	Population	Methods	Results	Authors’ Conclusions
Rodrigo L. et al., 2013 [68]	Prospective study	1 year GFD observation	229 patients: 125 (54%) with IBS, 104 were female (84%); 104 (46%) with IBS + FM, 93 were female (89%), 7 of the 104 IBS + FM patients had CD (7%)	Parameters assessed at baseline and after 1 year of GFD. Score examined: tender points (TPs) test, Fibromyalgia Impact Questionnaire (FIQ), Health Assessment Questionnaire (HAQ), Short Form Health Survey (SF-36), Visual Analogue Scales (VAS). Any changes in gastrointestinal complaints, pain and tiredness, drug prescriptions, and anti-tTG2 serum levels were recorded	At baseline, all patients had poor QoL and VAS scores, a high number of TPs and drug prescriptions, and increased tTG levels. After 1 year of GFD, all outcome measures significantly improved, with a decrease of 51–60% in TPs, FIQ, HAQ, and VAS scales, and in the number of prescribed drugs, accompanied by an increase of 48–60% in SF-36 Physical and Mental Component Summary scores, and a decrease in tTG2 to normal values	The adherence to a GFD by CD-related IBS/FMS patients can simultaneously improve CD and IBS/FMS symptoms
Rodrigo L. et al., 2014 [64]	Case-control study	1 year GFD observation	97 IBS + FMS females: 58 had duodenal intraepithelial lymphocytosis (Marsh stage 1), and 39 had a normal duodenal biopsy (Marsh stage 0)	Parameters assessed at baseline and after 1 year of GFD. Score examined: Fibromyalgia Impact Questionnaire (FIQ), the Health Assessment Questionnaire (HAQ), tender points (TPs), the Short Form Health Survey (SF-36), and the Visual Analogue Scales (VAS) for gastrointestinal complaints, pain, and fatigue	At baseline, all patients had a poor QoL and high VAS scores. After one year on a GFD, all outcome measures were better in the Marsh stage 1 group, with a mean decrease of 26 to 29% in the TPs, FIQ, HAQ and VAS scales, accompanied by an increase of 27% in the SF-36 physical and mental component scores. However, in the IBS plus FMS/Marsh stage 0 group, the GFD had almost no effect	GFD in the duodenal intraepithelial lymphocytosis-related IBS/FMS subgroup of patients can produce a slight but significant improvement in all symptoms
Isasi C. et al., 2014 [69]	Clinical report	5 to 31 months of GFD	20 patients with FM and duodenal intraepithelial lymphocytosis. CD was ruled out; they had clinical response to a GFD	Clinical response was defined as the achievement of at least one of the following scenarios: remission of FM pain criteria, return to work, return to normal life as judged by the patient, or opioid discontinuation	Eleven patients carried either the DQ2 or DQ8 heterodimers. Seven patients had only one allele of the DQ2 heterodimer. Two patients did not carry either DQ2 alleles or DQ8. The mean follow-up period for the gluten-free diet was 16.4 months. The level of chronic pain improved for all patients; for 15 patients, chronic widespread pain was no longer present. Fifteen returned to work or normal life. Three patients discontinued opioid treatment Fatigue, gastrointestinal symptoms, migraine, and depression also improved. Patients with oral aphthae, went into complete remission for psoriatic arthritis and undifferentiated spondylarthritis	Remarkable clinical improvement can be achieved with a GFD in patients with FM, suggesting that NCGWS may be an underlying treatable cause of FM syndrome. The presence of intraepithelial lymphocytosis in the duodenal biopsies of these selected patients further supports this hypothesis
Slim M. et al., 2017 [65]	Randomized clinical trial	FM patients were randomly allocated to receive a GFD or a HCD over a 24-week period	75 adults diagnosed with FM. GFD (*n* = 35) HCD (*n* = 40)	Primary outcome: change in the number of gluten sensitivity symptoms. Secondary outcomes evaluated body mass index, Revised Fibromyalgia Impact Questionnaire, Pittsburgh Sleep Quality Index, Brief Pain Inventory, Beck Depression Inventory-II, State-Trait Anxiety Inventory, Short-Form Health Survey, Patient Global Impression Scale of Severity, Patient Global Impression Scale of Improvement, and adverse events	Gluten sensitivity symptoms did not differ significantly between the GFD and HCD (GFD −2.44 ± 0.40; HCD −2.10 ± 0.37; *p* = 0.343). Two dietary interventions did not differ in any of the remaining measured secondary outcomes (FM-related symptoms questionnaires). Both dietary interventions were well tolerated	GFD and HCD have both beneficial outcomes in reducing gluten sensitivity symptoms and other secondary outcomes. GFD was not superior to HCD in reducing the number of gluten sensitivity symptoms or secondary outcomes

FM: fibromyalgia; GFD: gluten free diet; HCD: hypocaloric diet; tTG2: tissue transglutaminase.

## 8. Autoimmune Thyroiditis

The rationale behind the application of a GFD in autoimmune thyroiditis lies in the association between celiac disease and other autoimmune diseases. Indeed, as stated by the “leaky gut” theory [9], the increase in intestinal permeability, possibly triggered by gluten ingestion, leads to autoimmunity in predisposed patients. Chronic autoimmune thyroiditis (CAT) has a higher prevalence in females, and the coexistence of CD and CAT ranges between 2–7.8% [70]. Nevertheless, the general assessment of a link between the direct effect of gluten exposure and the development of autoimmune disorders is far from granted [71].

A recent metanalysis [72] summarized the result of four studies regarding the effect of a GFD on anti-thyroglobulin antibodies (TgAb), anti-thyroid peroxidase antibodies (TPO), TSH, ft3, and ft4 levels in patients with CAT and no symptoms or histology of CD. The statistical analyses highlight a decrease in TgAb and TPoAb, despite a wide variation of the values being found. A statistically significant improvement is claimed for both TSH and ft4. In contrast, the results reported in the single studies considered are heterogenous, and are briefly discussed in the next paragraphs and in Table 2.

Krysiak et al. (2019) [73] found a significant reduction in anti-TPO and anti-TG after 6 months of GFD in patients with CAT, but TSH and free thyroid hormones were not affected. The authors suggest a possible beneficial effect in euthyroid CAT women, which might prevent the development of hypothyroidism. 

**Table 2:** Experimental design studies relating chronic autoimmune thyroiditis and gluten-free diet.

Authors	Type of Study	Duration of GFD	Population	Methods	Results	Authors Conclusions
Poblocki J. et al., 2021 [74]	Randomized clinical trial	12 months of GFD vs. any dietary treatment	62 Caucasian women with CAT. GFDG (*n* = 31); CG (*n* = 31)	Serum concentrations of TSH, ft3, ft4, anti-TPO, and anti-TG2 were determined in all patients at baseline, after 3, 6, and 12 months of observation	No differences were found in anti-TPO and anti-TG antibodies or ft3 and ft4 levels, except a significant reduction in TSH (*p* < 0.044) levels in the GFDG. Analysis between appointments presented no significant differences in changes in concentrations of anti-TPO, anti-TG, or ft3, but after analyzing the changes in the median concentration of the tested blood indices, a significance was noticed in TSH (*p* = 0.039) and fT4 (*p* = 0.022). An analysis of changes in the concentration of the studied parameters after logarithmic transformation was also performed, which showed the improvement in anti-TG, TSH, and ft4 at 3, 6, and 12 months of the intervention	There are no clear indications to routinely follow a GFD because of CAT, and it is necessary to perform more studies to assess if CAT patients achieve the benefits of following a GFD
Krysiak R. et al., 2019 [73]	Prospective nonrandomized study	6 months of GFD vs. any dietary treatment	34 women with CAT but euthyroid. GFDG *n* = 16, CG *n* = 18. Incidentally found that positive anti-tissue transglutaminase antibodies without clinical symptoms of coeliac disease was an inclusion criteria	Serum titers of anti-TPO and anti-TG, TSH, ft3, ft4, 25-hydroxyvitamin D were measured. Based on thyrotropin and free thyroid hormone levels, Jostel’s thyrotropin index, the SPINA-GT index, and the SPINA-GD index were calculated.	CG: serum TSH and ft3, ft4 levels, serum 25-hydroxyvitamin D level, and calculated indices remained at the similar levels. GFDG had reduced thyroid antibody titers, slightly increased 25-hydroxyvitamin D levels and the SPINA-GT index. In GFDG, the impact on TPOAb and TgAb titers correlated with the changes in the SPINA-GT index, whereas the impact on TPOAb correlated with the changes in 25-hydroxyvitamin D levels	The GFD reduced serum titers of TPOAb and TGAb in euthyroid women with CAT, which correlated with the increase in the SPINA-GT index. This finding indicates that the GFD may bring clinical benefits to euthyroid women with CAT, who, because of markedly elevated thyroid antibody titers, are at high risk of the development of hypothyroidism
Krysiak R. et al., 2022 [75]	Pilot study, case-control study	12 months of GFD + vit D supplementation vs. no dietary intervention + vit D supplementation	31 NCGWS woman with CAT vs. 31 women matched for thyroid antibody titers	Anti-TPO, Anti-TG, plasmatic TSH, ft3, ft4, prolactin, 25-hydroxyvitamin D, and CRP were measured at entry and after a 6-month follow-up	In the CG, a significant decrease in anti-TPO (*p* = 0.0017) and anti-TG (*p* = 0.0056) level, and an increase in vitamin D concentration (*p* = 0.0006), were registered, compared with the GFDG. Although the typical diet showed better results in improving anti-TPO anti-TG and vitamin D than the GFD, in both groups a significant improvement in these parameters was observed. No changes were observed in the case of TSH, fT3, and fT4 in the groups and between them	The obtained results suggest that GFD may impair beneficial effects of exogenous vitamin D in individuals with CAT

TPO: thyroid peroxidase; TG: thyroglobulin; CRP: C reactive protein; CAT: chronic autoimmune thyroiditis; Ab: antibodies; GFDG: gluten-free diet group; CG: control group; QoL: quality of life.

Pobłocki et al. [74] highlighted an amelioration of TSH and ft4 only after logarithmic transformation, at the end of 12 months of GFD in CAT patients. They conclude that a GFD cannot be recommended in CAT patients.

Krysiak R. et al. (2022) [75] examined the effect of GFD plus vitamin D supplementation in women with CAT and NCGWS. No changes in TSH, ft3 and ft4 were found, and only the group following a gluten containing diet plus vitamin D showed a decrease in anti-TPO and anti-TG levels. The hypothesis of the authors is that GFD can reduce the beneficial effects of vitamin D supplementation. 

Abbott et al. [76] analyzed a wider dietary and lifestyle approach including the elimination of legumes, nightshades, dairy, eggs, coffee, alcohol, nuts, seeds, refined/ultra-processed sugars, oils, and food additives in addition to gluten, giving patients online support. The latter might be misleading for the results of the metanalysis for the presence of several variables and not only for the adherence to a GFD [72].

In conclusion, there are several limitations regarding the scarcity of the literature on the topic, the small sample size of the studies, composed only of women, and their heterogeneity (some studies included patients with positive anti-tissue transglutaminase antibodies or NCGWS; others ruled out CD). To date, it is not possible to claim a beneficial effect of a GFD in CAT, and more studies are needed to shed light on this topic. Moreover, it should be kept in mind that a GFD is often linked to an inadequate intake of fats, proteins, sodium, and vitamins, and it should be prescribed only when a clear beneficial effect is acknowledged [77].

## 9. Endometriosis

Endometriosis is characterized by the presence of endometrial-like tissue functioning outside the uterus, affecting 5–10% of women of reproductive age globally. It is a challenging disease, often misdiagnosed, that constitutes a burden for patients affected, due to its social impact and the coexistence of other conditions such as fibromyalgia, migraines, IBS [78]. Indeed, studies analyzing health-related QoL of women suffering from endometriosis correlates the disease with an increased incidence of depression and anxiety, especially related to chronic pain [79]. Moreover, therapies currently available are in most cases inadequate for a proper management of symptoms [78].

For these reasons, many women seek non-medical intervention to relieve pain and improve their QoL such as exercise, meditation, and dietary changes [80,81]. A cross-sectional study by Mazza et al. [82] developed an online questionnaire monitoring self-reported changes in eating habits after an endometriosis diagnosis. Of 4078 women, 17% reported having autoimmune disease, and, more specifically, CD was reported by 1.2% of the population. Among the total study population, a percentage of 66.4% of women changed their dietary habits, with the majority choosing a GFD (15%). As stated by the authors, these findings are probably indicating an attempt to manage inflammation, especially in stage IV disease. The choice of avoiding gluten might be sustained by an increased awareness of the impact of nutrition on health. To date, we found only a retrospective study [83] (Table 3) that investigated GFD as a tool for the management of endometriosis-related pain and QoL. The research was conducted on 207 women on a GFD for 12 months. Three-quarters of patients (156 subjects, 75%) reported a statistically significant change in painful symptoms (*p* < 0.005), and an amelioration of all domains of physical functioning, general health perception, vitality, social functioning, and mental health was observed in all patients (*p* < 0.005). 

Thus, it seems clear that diet management in patients with endometriosis is a topic that needs to be properly addressed [81]. Despite the encouraging results of the study here presented, the scarceness of higher-quality and larger sample studies represents an insurmountable limit that sets us far back from prescribing a GFD in endometriosis [84]. Moreover, pathogenetic hints on the reason why a GFD should be beneficial are lacking.

## 10. Conclusions

A GFD is often erroneously considered both a healthy lifestyle choice and an option for the management of different diseases, particularly by women [13,14]. De facto, without a nutritional supervision, a GFD tends to be unbalanced in its caloric and nutritional values. Moreover, it is usually more expensive and socially impactful, and therefore hard to follow.

Despite the presence of hints that might suggest the involvement of gluten or other wheat compounds (such as ATIs) in the development of AD or other diseases involving a dysregulation of the immune system, GFD is not for everyone. The only condition that requires a strict adherence to gluten avoidance is CD. NCGWS patients benefit from a gluten restriction, which might not be lifelong. 

The literature summarized in this study is not sufficient to suggest the application of a GF regime as a dietary treatment for FM, CAT, and endometriosis. The experimental design studies relating the application of a GFD were only (1) four for FM [64,65,68,69], (2) three for CAT [73,74,75], (3) one for endometriosis [83]. Therefore, there is a need for higher-quality studies with a proper stratification of the patients to better define a role for gluten in the management of different pathological conditions. Moreover, there is a concrete risk of nutritional imbalance, especially when professional support is not provided. 

Instead, according with the experience derived from NCGWS studies, we can suggest looking for NCGWS in patients with FM. Also, we suggest that in patients with AD, the presence of both CD and NCGWS should be evaluated. Only in the case of the coexistence of gluten-related disorders do we recommend gluten restriction, here proven to be beneficial. 

## Figures and Tables

**Table 3:** Experimental design study relating endometriosis and gluten-free diet.

Authors	Type of Study	Duration of GFD	Population	Methods	Results	Author’s Conclusions
Marziali M. et al., 2012 [83]	Retrospective study	1 year of GFD	207 women with severe painful endometriosis-related symptoms	Baseline values of painful symptoms were assessed by Visual Analogue Scale (VAS) for dysmenorrhea, nonmenstrual pelvic pain, and dyspareunia, and re-evaluated after 12 months	At 12-month follow-up, 156 patients (75%) reported statistically significant change in painful symptoms (*p* < 0.005), 51 patients (25%) reported any improvement of symptoms. No patients reported worsening of pain. A considerable increase in scores for all domains of physical functioning, general health perception, vitality, social functioning, and mental health was observed in all patients (*p* < 0.005)	Painful symptoms of endometriosis decrease after 12 months of GFD

GFD: gluten-free diet.
